# GERIATRIC WORKFORCE TRAINING TO ADVANCE AGE-FRIENDLY CARE IN RURAL COMMUNITIES

**DOI:** 10.1093/geroni/igae098.0063

**Published:** 2024-12-31

**Authors:** Jennifer Severance, Sara Murphy

**Affiliations:** Chair; Co-Chair

## Abstract

Texas has one of the highest rates of rural hospital closures and a scarcity of healthcare professionals, including primary care physicians and registered nurses, with 82% of Texas counties designated as primary care shortage areas. Critical workforce shortages require innovative training models. To address challenges in rural communities across Texas and the surrounding region, a HRSA-funded Geriatrics Workforce Enhancement Program (GWEP) leveraged partnerships with academic, primary care, long-term care, and community-based organizations and expertise in geriatric clinical care. This symposium explores innovative approaches to geriatrics training by sharing critical resources, responding to community needs, and applying technology to transform clinical training environments for medical students, primary care residents, practicing health care teams, and older adults and caregivers in rural communities; expand organizational capacity to provide quality geriatrics care; and replicate evidence-based practices to impact older adults at the individual, community, and population levels. First, presenters will provide an overview of the overall strategy involving collaborators as key connections and subject matter experts for the unique needs and opportunities in rural communities. This is followed by individual projects engaging medical students, residents, practitioners, and older adults and caregivers as learners and change agents for Age-Friendly care. This symposium presents cross-sector recruitment strategies using technology and partnerships as well as methods of adapting these models to a local context while expanding training opportunities for rural areas and underserved populations.

